# Supplementary material for: A Novel Mouse Monoclonal Antibody C42 against C-Terminal Peptide of Alpha-1-Antitrypsin
Source: Int J Mol Sci. 2021 Feb 21;22(4):2141. doi: 10.3390/ijms22042141 (PMC7926790; doi:10.3390/ijms22042141)
Supplement: Supplementary file 1 [file ijms-22-02141-s001.pdf]

Supplementary Figure 1

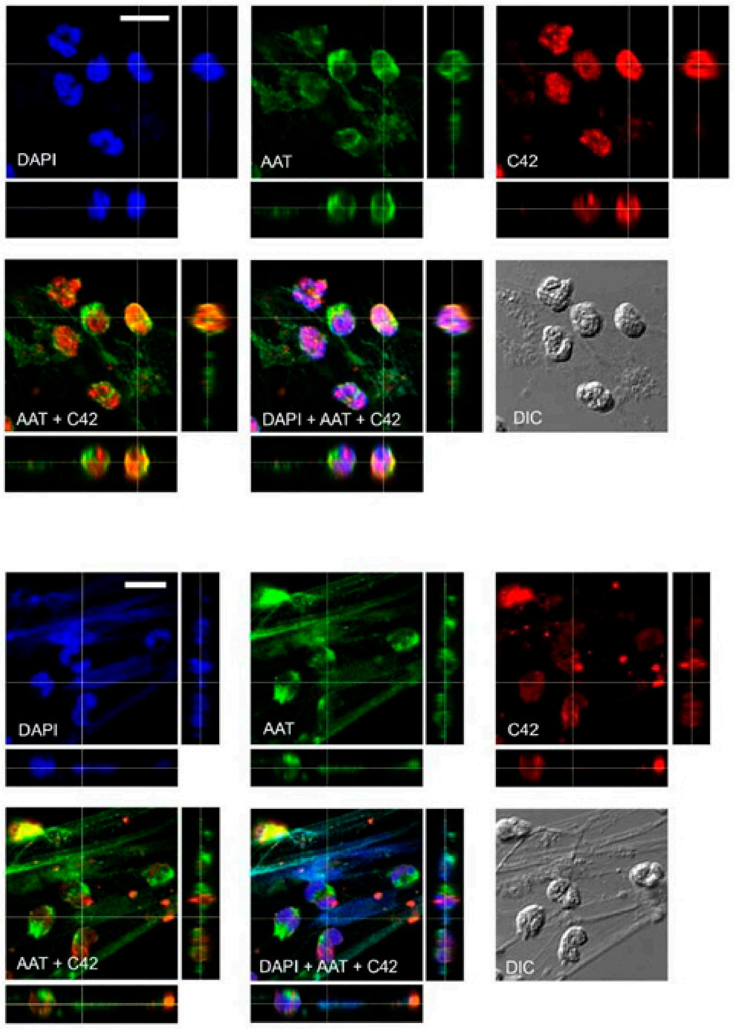

Suppl. Figure 1. Localization of the C-36 peptide and full-length AAT in NETs formed by LPS-stimulated human neutrophils as visualized in series of optical cross-sections of fine (A) and coarse (B) NETs meshwork.

(A) Confocal z-stacks, corresponding to the image in Fig. 6, reveal noticeable segregation between C-36 (*red*) and AAT (*green*) signals in neutrophils as well as in the fine NETs structures formed upon LPS stimulation. Full-length AAT strongly associates with the thin meshwork of DNA fibers and accumulates at the periphery of the cells trapped in the NETs. The C-36 is predominantly present in perinuclear and probably nuclear compartments of the cells. The C-36-positive aggregates trapped in the NETs meshwork were also visible. DAPI fluorescence (*blue*) on the fine DNA fibers of the NETs meshwork was substantially weaker than the fluorescence of the nuclei. Since dynamic range of the photomultiplier did not cover well such dramatic difference in intensity, we compensated it by overexposing nuclei in the blue channel to visualize the fine DNA fibers (see Fig. 6, last panel). (B) Distinct patterns of the C-36- and AAT-positive signals observed in well-developed NETs. The AAT signal colocalizes with DNA fibers and is detectable as patches in remaining cells. Aggregates strongly positive for C-36 do not colocalize with the DNA and AAT fibers, but appear to associate with them. Relatively weak C-36 signal is also detectable in the cell remains. DIC, differential interference contrast. Scale bar, 10 µm.
